# Lipophilic statins limit cancer cell growth and survival, via involvement of Akt signaling

**DOI:** 10.1371/journal.pone.0197422

**Published:** 2018-05-15

**Authors:** Colin H. Beckwitt, Keisuke Shiraha, Alan Wells

**Affiliations:** 1 Pathology, University of Pittsburgh, Pittsburgh, PA, United States of America; 2 The University of Pittsburgh Cancer Institute, University of Pittsburgh, Pittsburgh, PA, United States of America; 3 Pittsburgh VA Health System, Pittsburgh, PA, United States of America; 4 Bioengineering, University of Pittsburgh, Pittsburgh, PA, United States of America; 5 Computational and Systems Biology, University of Pittsburgh, Pittsburgh, PA, United States of America; University of Manitoba, CANADA

## Abstract

The HMG-CoA reductase inhibitors, statins, have been used as lipid lowering drugs for decades and several epidemiological studies suggest statin usage correlates with a decreased incidence of cancer specific mortality in patients. However, the mechanism of this mortality benefit remains unclear. Here, we demonstrate that statin drug lipophilicity and affinity for its target enzyme, HMGCR, determine their growth suppressive potency against various tumor cell lines. The lipophilic atorvastatin decreases cancer cell proliferation and survival *in vitro*. Statin sensitivity coincided with Ras localization to the cytosol instead of the membrane, consistent with a decrement in prenylation. To investigate signaling pathways that may be involved with sensitivity to statin therapy, we employed inhibitors of the PI3K-Akt and Mek-Erk signaling cascades. We found that inhibition of PI3K signaling through Akt potentiated statin sensitivity of breast cancer cells *in vitro* and in co-culture with primary human hepatocytes. The same effect was not observed with inhibition of Mek signaling through Erk. Moreover, the sensitivity of breast cancer cells to atorvastatin-mediated growth suppression correlated with a decrease in EGF-mediated phosphorylation of Akt. As an increase in Akt activity has been shown to be involved in the metastasis and metastatic outgrowth of many cancer types (including breast), these data suggest a mechanism by which statins may reduce cancer specific mortality in patients.

## Introduction

Cancer is the second highest cause of mortality in the United States despite many advances made in therapeutic development and clinical management [1]. Nearly all cancer deaths can be attributed to metastatic disease. The metastatic cascade concludes with the establishment of micrometastases at the target distant organ site [2]. Distant micro-metastases bear poor prognosis for cancer patients, which is due in part to clinically silent cells that only outgrow to form clinically apparent metastases after periods of dormancy which can last years to decades [3]. Preventing metastasis or subsequent outgrowth would delay this major cause of cancer mortality. Unfortunately, by the time the primary tumor has been found, many tumor cells may have already disseminated to distant sites and established dormant micrometastases [4]. The clinical challenge in targeting dormant micrometastases is that their quiescent cells exhibit chemoresistance to many available standard therapies, which mostly target dividing cells [5]. Therefore, there is a great need for alternative therapies that either prevent metastasis initiation or suppress micrometastatic emergence.

Since the development of new therapies is quite costly, taking years to decades for new drugs to be implemented in a clinical setting, repurposing existing drugs with favorable safety profiles presents an opportunity to uncover new approaches that may be beneficial in metastatic disease [6,7]. The HMG-CoA reductase (HMGCR) inhibitors, statins, have been clinically approved for the treatment of dyslipidemias for several decades [8]. Large retrospective cohort studies of cancer patients taking statins for other conditions have uncovered that their use appears to reduce cancer mortality, particularly in breast cancer [9,10] while having no consistent influence on cancer incidence [11,12]. These clinical data have been reinforced by cell and animal data demonstrating statins exhibit anti-tumor effects by inducing apoptosis or growth arrest [13–18]. However, not all cancer cells are sensitive to statin therapy and prospective clinical trials remain inconclusive [19]. We propose that the divergence relates to limited understanding of the cellular and molecular mechanisms of actions of statins on difference cancer stages.

HMGCR acts at the rate-limiting step in the cholesterol biosynthesis pathway by catalyzing the conversion of HMG-CoA to mevalonic acid. Important byproducts of this pathway, aside from cholesterol, include the isoprenoid intermediates geranyl-geranyl pyrophosphate and farnesyl pyrophosphate, whose attachment to small signaling G-proteins, including Ras, Rho, and Rac, is critical to their functioning [20,21]. Several clinical studies of statins’ usage in cancer patients have suggested lower cancer mortality and recurrence risk in patients using lipophilic statins when compared to those on hydrophilic statins [22,23]. In vitro studies have shown lower anti-tumor effects using hydrophilic pravastatin when compared with lipophilic simvastatin due to the lack of facilitated uptake by the apical transporter SLCO1B1 that is expressed endogenously in liver tissue [13]. However, pravastatin also has an order of magnitude lower affinity for the target enzyme, HMGCR [24].

We previously demonstrated that multiple tumor cell lines exhibit differential relative sensitivities to atorvastatin [14]. Parenthetically, we define statin sensitivity as a relative term, which quantitatively corresponds to an IC50 to atorvastatin less than 5μM *in vitro*. Importantly, we determined that membrane E-cadherin (E-cad) indicates resistance to statin therapy [14]. E-cadherin inhibits proliferation by attenuating the Hippo signaling pathway [25], which may decrease the need for the rapid lipid biosynthesis commonly seen in rapidly proliferating cells [26]. However, the nature of E-cadherin-mediated statin resistance and the cellular response of tumor cells to statins, including the signaling pathways involved, are still not well characterized. Here, we now show that statin pharmacologic properties govern efficacy against cancer cell lines. We find that rosuvastatin, a hydrophilic statin with similar HMGCR affinity to atorvastatin’s, is less potent at suppressing tumor cell growth, while pravastatin, a hydrophilic statin with a tenfold lower affinity, has no influence on tumor cell growth. We also demonstrate that atorvastatin mediated growth suppression is due to a combination of a reduction in both the proliferation and survival of tumor cells. Moreover, statin treatment promotes cytoplasmic localization of Ras, whose localization to the membrane is critical for activity [20]. To probe the signaling pathways that are involved with susceptibility to statins, we demonstrate that statin treatment suppresses cellular signaling through two canonical survival pathways, that of Akt and Erk, both basally and under stimulation by EGF. Both Erk and AKT are activated downstream of Ras via intermediary kinases [27,28], and thus could be affected by the statin-induced reduction in prenylation. Most significantly, signaling through Akt was profoundly affected by statin treatment and was potentiated by concurrent inhibition of PI3K, a target of Ras activation [27]. As dysregulation of PI3K-Akt signaling occurs with EMT, invasion, and metastasis [29], these data may suggest one mechanism by which statins act to reduce mortality in breast cancer patients.

## Materials methods

### Cell sourcing and cell culture

All cell lines were sourced directly from the ATCC. Cell lines derived from the following tumor primary sites were employed: breast (MCF-7 and MDA-MB-231), prostate (DU-145), brain (SF-295), and melanoma. DU-L and DU-H describe DU-145 cells, both directly sourced from the ATCC, with low and high levels of E-cadherin respectively, and are described more comprehensively in the following reference [30]. MCF-7 RFP, MDA-MB-231 RFP, MDA-MB-231 RFP/Ecad, and DU-145 RFP were previously developed in our lab by stable transfection of RFP [2]. All cells were maintained in RPMI 1640, GlutaMax Supplement (Gibco, ThermoFisher Scientific) supplemented with 10% HI-FBS (Gemini Bioproducts) and 0.5% penicillin-streptomycin (Gibco, ThermoFisher Scientific), henceforth referred to as RPMI. Stably transfected RFP or RFP/Ecad cells were maintained with additional supplementation of puromycin (Gibco, ThermoFisher Scientific) or G418 (Teknova) at the following concentrations: 1μg/mL puromycin (MCF-7 RFP), 5μg/mL puromycin (MDA-MB-231 RFP), 900μg/mL G418 (MDA-MB-231 RFP/Ecad and DU-145 RFP). Antibiotic selection media was removed prior to beginning experiments. Primary human hepatocytes were obtained as isolates from excess pathology specimens at UPMC as part of an NIDDK-funded Liver Tissue and Cell Distribution System run by Dr. David Gellar funded by NIH contract #HHSN276201200017C. Hepatocytes were isolated by collagenase perfusion for subsequent distribution to investigators.

### Chemicals

Atorvastatin (PHR-1422), rosuvastatin (SML-1264), pravastatin (P4498), and simvastatin (S6196) were all obtained from Sigma Aldrich, USA. Atorvastatin and rosuvastatin were dissolved in DMSO at a concentration of 50mM. DMSO was used for both statins to maintain carrier consistency between the two primary statins used for this work. Pravastatin was dissolved in sterile milli-Q water at a concentration of 50mM. Simvastatin lactone was dissolved in 200-proof EtOH at a concentration of 50mM. Activated simvastatin was prepared by dissolving simvastatin lactone at a concentration of 50mg/mL in 200-proof EtOH. 1N NaOH was added to a final concentration of 450mN and the solution was heated in a 50°C water bath for 2 hours. After heating, the solution was stored in aliquots and frozen. Prior to treatment, the pH was lowered to 7.4 by adding 1N HCl. Human EGF (E9644, Sigma, USA) was reconstituted in 1% BSA in normal saline at a concentration of 1mM. PD98059 (S1177, SelleckChem, USA) was reconstituted in DMSO at a concentration of 20mM. LY294002 (S1105, SelleckChem, USA) was reconstituted in DMSO at a concentration of 10mM.

### Statin IC_50_ determination

Cells were seeded in 24-well plates at a concentration of 5x10^4^ cells/mL in a volume of 500μL. The next morning, cells were treated with atorvastatin, rosuvastatin, pravastatin, or simvastatin in half log doses between 100nM and 100μM. The vehicle control treatment used for each statin was 0.2% DMSO for atorvastatin and rosuvastatin, 0.2% EtOH for simvastatin, or complete media for pravastatin. For co-treatment with PD98059 or LY294002, statin solutions were prepared in solutions of the desired PD98059 or LY294002 concentration. After 72 hours of treatment, treatment solutions were aspirated and cells were fixed with 3.7% formaldehyde (F79-1, ThermoFisher Scientific) for 15 minutes. After fixation, cells were incubated with 0.5% w/v crystal violet for 10 minutes and excess dye was removed by copious washing with tap water. The absorbed dye was released with 2% SDS and mixed thoroughly before transferring to a 96 well microplate and reading at 560nm using a Tecan SpectraFluor microplate reader (Tecan US, Durham, NC). IC_50_ values were determined by fitting a standard, four-parameter sigmoid curve to the data. All treatments were carried out in triplicate samples and all data are representative of at least three independent experiments.

### Transfection

MDA-MB-231 RFP cells were seeded in 6-well plates at a concentration of 1.5x10^5^ cells/mL in a volume of 2mL. The next morning, cells were transfected in 2mL Optimem (Gibco, ThermoFisher Scientific) with 10μL/well of Lipofectamine RNAi-Max (ThermoFisher Scientific) and either 20nM non-coding (NC) siRNA (Silencer Select Negative Control No. 1 siRNA, Cat # = 4390843, ThermoFisher Scientific) or 20nM HMGCR siRNA (Silencer Select siRNA, siRNA ID = 141, Cat# = 4392420, ThermoFisher Scientific). Four hours after transfection, media was changed to complete media for two hours to allow cells to recover. Cells were then seeded in 24-well plates as per the “Statin IC_50_ Determination” protocol above to determine the IC50 of the cells to atorvastatin, pravastatin, and doxorubicin (APP Pharmaceuticals LLC).

### Statin proliferation assay

MCF-7 RFP, MDA-MB-231 RFP, or MDA-MB-231 RFP/Ecad cells were seeded in 12-well plates on heat-sterilized glass coverslips (Cat# 12-545-80, ThermoFisher Scientific) at a concentration of 1.5x10^5^ cells/mL in a volume of 1mL. The next morning, cells were treated with atorvastatin or rosuvastatin at doses of 1μM, 5μM, 20μM, or 60μM for 48 hours. Cells treated with 0.12% DMSO served as the vehicle control. After 24 hours of treatment, concentrated EdU (ThermoFisher Scientific) was added to each well to a final concentration of 10μM. After 24 hours of EdU treatment (48 hours of statin treatment), treatment solutions were aspirated and cells were fixed with 3.7% formaldehyde (F79-1, ThermoFisher Scientific) for 15 minutes and stained for EdU as described below.

### EdU staining

Cells fixed on coverslips were permeabilized with 0.1% Triton-X-100 (ThermoFisher Scientific) for 20 minutes then washed once with 3% Bovine Serum Albumin (Sigma). Cells were stained for EdU using the Click-iT EdU Alexa Fluor 488 Imaging Kit (ThermoFisher Scientific) per the manufacturer’s instructions. After EdU staining, the cells were counter-stained with 2.5μg/mL DAPI for 15 minutes, washed three times in normal saline, and mounted using a glycerol and PVA based mounting medium courtesy of the Center for Biological Imaging at the University of Pittsburgh. Coverslips were allowed to harden overnight prior to imaging.

### Statin survival assay

MCF-7 or MDA-MB-231 cells were seeded in 12-well plates at a concentration of 1.5x10^5^ cells/mL in a volume of 1mL. The next morning, cells were treated with 1μg/mL propidium iodide and either complete media (control), 1μM doxorubicin, or 5μM atorvastatin for 72 hours. Cells were imaged on an inverted microscope (Olympus, Model IX70) using a 10x objective to capture both phase contrast and red fluorescence images (561nm) 0, 24, 48, and 72 hours after treatment.

### Cytoplasmic and membrane protein extraction

Cells were harvested using 0.25% Trypsin (ThermoFisher Scientific) and quenching with RPMI. Cells were spun at 1000 RPM for 4 minutes and washed once with 1.5mL ice cold PBS with Calcium and Magnesium (Corning). Cells were respun at 1000 RPM for 4 minutes and the supernatant was carefully aspirated. The cell pellet was resuspended in 50μL 0.2% w/v Saponin (Sigma) supplemented with 1:100 protease inhibitor cocktail V (CalBioChem) and incubated for 15 minutes on ice. After incubation, the membrane was pelleted by centrifuging at 13000g for 15 minutes at 4°C using a refrigerated microcentrifuge (Savant, SFR13K). The supernatant was carefully separated from the membrane pellet and labeled as the cytoplasmic protein fraction. The membrane pellet was washed twice with 1.5mL of 0.2% saponin, centrifuging the pellet after each wash by spinning at 13000g for 15 minutes at 4°C. Extra washing steps of the membrane were performed to further purify the protein that remained membrane-tethered after initial saponin extraction of the cytoplasm. After the second wash, the membrane protein was eluted by adding ice cold 50μL RIPA buffer (50mM Tris-HCl, 150mM NaCl, 1mM EDTA, 0.1% SDS, 0.5% deoxycholate, 1% NP-40, pH 8.0) supplemented with 1:100 protease inhibitor cocktail V and incubating for 15 minutes on ice. After incubation, the samples were sonicated for 2 seconds (BioLogics Inc., Model 150 V/T) and centrifuged at 13000g for 10 minutes at 4°C. The supernatant was carefully separated from the pellet and labeled as the membrane protein fraction.

### Phospho-protein extraction

Cells were lysed using RIPA buffer supplemented with 1:100 protease inhibitor cocktail V (CalBioChem, USA) and 1mM Na_3_VO_4_ and collected into Eppendorf tubes using a cell scraper. The samples were sonicated for 2 seconds and centrifuged at 13000g for 10 minutes at 4°C. The supernatant was carefully separated from the pellet into a new tube for sample preparation.

### Active Ras analysis

The Ras Activation Assay Kit (Cat# 17–218, Millipore Sigma, USA) was used to pull down Ras-GTP using agarose beads GST-tagged with the Raf-1 Ras binding domain (Raf-1 RBD), per the manufacturer’s instructions. MDA-MB-231 RFP cells were treated with or without 1μM atorvastatin for 48 hours and then cells were stimulated with 5nM EGF for 5 minutes. Cells were lysed using the kit-provided lysis buffer, supplemented with 1:100 protease inhibitor cocktail V (CalBioChem, USA), and the lysates were collected into Eppendorf tubes using a cell scraper. The samples were sonicated for 2 seconds and centrifuged at 13000g for 10 minutes at 4°C. The supernatant was carefully separated from the pellet into a new tube for sample preparation. The supernatants were incubated with Raf-1 RBD agarose beads for 45 minutes at 4°C with continuous gentle mixing. After incubation, beads were pelleted at 14000g for 10 seconds, and washed three times with lysis buffer. After the third washing step, beads were resuspended in loading buffer, boiled for 5 minutes, and processed per the “Western Blotting” protocol below, taking care to centrifuge agarose beads prior to sample loading. The ‘active’ form runs at a mobility slightly faster than the inactive form which is also pulled down though less efficiently.

### Western blotting

Protein concentration was determined by the BCA Protein Assay Kit (Pierce, ThermoFisher Scientific). Samples were prepared and boiled for 5 minutes prior to loading. Proteins were separated on 9% or 15% Tris Bis-acrylamide gels prepared the same day at 96V (E-C Apparatus Corp., EC-105) until adequate separation was achieved. Samples were transferred onto nitrocellulose membrane at room temperature at 300mA for 1.5 hours (E-C Apparatus Corp., EC135-90). After transferring, membranes were blocked in 5% w/v non-fat dry milk in tris buffered saline with 0.5% Tween-20 (TBS-T). Membranes were probed with primary antibodies in 5% w/v non-fat dry milk overnight at 4°C on a rotator. The primary antibodies used were anti-Pan Ras (1:1000, MA1-012X, ThermoFisher Scientific), anti-MHC-I (1:500, sc-55582, Santa Cruz Biotech), anti-GAPDH (1:20000, G9545, Sigma), anti-E-cadherin (1:1000, 3195S, Cell Signaling), anti-HMGCR (1:1000, ab174830, Abcam), anti-Pan Akt (1:1000, 4691S, Cell Signaling), anti-Erk1/2 (1:2000, 4695S, Cell Signaling), anti-Phospho-Akt (1:1000, 4060S, Cell Signaling), and anti-phospho-Erk1/2 (1:2000, 4370S, Cell Signaling). After primary incubation, membranes were washed 3 times for 10 minutes each in TBS-T. Species-specific horseradish-peroxidase conjugated secondary antibodies were applied at room temperature in 5% w/v non-fat dry milk for 1 hour. The two secondary antibodies used were anti-rabbit IgG (1:5000, Sigma, A9169) and anti-mouse IgG (1:5000, Sigma, A4416). After secondary incubation, membranes were washed 3 times for 10 minutes each in TBS-T. Membranes were incubated with ECL western blotting substrate (Pierce, ThermoFisher Scientific) and photo developed. Western blots were scanned at a resolution of 300 DPI and grayscale bit depth of 16. Bands were quantified using Image J software.

### Immunofluorescence microscopy

Single field fluorescent images were taken using an Olympus BX40 upright microscope with a 10x objective and fluorescence excitation wavelengths of 405 (DAPI), 488 (Click-iT EdU), and 561 (RFP). Exposures were kept identical across coverslips for each individual experiment for consistency. Image analysis was performed in NIS Elements version 4.5. Nuclei (DAPI) and proliferating nuclei (EdU) channels were labeled using spot detection for bright, clustered spots, and using the same parameters for all images associated with that experiment. Once individual channel masks were created, combined channel masks were generated by using the “having” command, which creates a new mask that illustrates all pixels of the first mask that contain at least one pixel of the second. This strategy was used to create a mask for DAPI + EdU (proliferating cell number). After generating all masks, data were measured and extracted for organization and presentation.

### Statistics

Statistics were conducted using GraphPad Prism 7 (Graphpad, USA). In all figures, the data are presented as the mean of three independent experiments, when counting the experiments were performed in triplicate, with the error bars representing the standard error of the mean. Comparisons of individual columns were determined by use of a student’s two-tailed unequal variance t-test and comparisons of dose curves were made using a two way analysis of variance without sample matching. Significance levels are reported in the figure legends and are kept consistent across all figures with symbols denoting * P < 0.05, ** P < 0.01, *** P < 0.001, and **** P < 0.0001.

## Results

### Statins suppress cancer cell growth differentially

**Fig 1:**
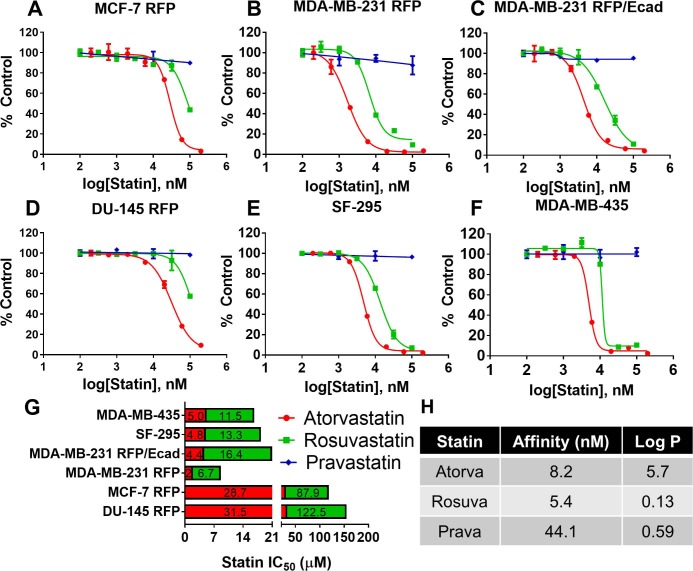
Lipophilic atorvastatin is more effective at suppressing cancer cell growth than hydrophilic rosuvastatin or pravastatin. Dose response curves for (A) MCF-7 RFP, (B) MDA-MB-231 RFP, (C) MDA-MB-231 RFP/Ecad, (D) DU-145 RFP, (E) SF-295, and (F) MDA-MB-435 cancer cells when cultured with atorvastatin (red), rosuvastatin (green), or pravastatin (blue) for 72 hours. Cell number was determined by crystal violet staining. Sigmoidal curves were fit to the dose response data and (G) IC_50_ values of atorvastatin and rosuvastatin in each cell line were extrapolated. (H) Pharmacologic parameters of atorvastatin, rosuvastatin, and pravastatin as found in the literature [24,31]. All data are representative of at least three independent experiments.

We previously demonstrated that atorvastatin could suppress the growth of many cancer cell lines [14]. We were curious whether other statins would show similar growth suppressive effects or whether growth suppression would be governed by the pharmacological properties of the specific statin. To compare to the lipophilic atorvastatin, we also tested rosuvastatin and pravastatin, both hydrophilic statins. While rosuvastatin shares a similar affinity for HMGCR as atorvastatin, pravastatin has approximately a 10-fold lower affinity for the enzyme (S1 Table). We found that atorvastatin was the most effective and pravastatin was the least effective at suppressing the growth of cancer cell lines, including breast (MCF-7 RFP, MDA-MB-231 RFP, and MDA-MB-231 RFP/Ecad, Fig 1A–1C), prostate (DU-145, Fig 1D), brain (SF-295, Fig 1E), and melanoma (MDA-MB-435, Fig 1F). Rosuvastatin was 2.3- to 3.9-fold less potent than atorvastatin while pravastatin showed no growth suppressive efficacy up to concentrations of 100μM. Moreover, simvastatin, a lipophilic and high affinity statin, demonstrated comparable efficacy to atorvastatin (S1 Fig). These results suggest that lipophilic, high potency statins are the most effective at suppressing tumor cell growth.

To confirm the growth suppressive effects of statins are due to its known mechanism of action (inhibition of HMGCR), we reduced HMGCR levels using siRNA transfection (S2 Fig). We found that a knockdown of 50% at the protein level in MDA-MB-231 breast cancer cells was sufficient to suppress cell growth and also potentiated the efficacy of both atorvastatin and pravastatin by over an order of magnitude (S2 Fig). In contrast, sensitivity to doxorubicin, a chemotherapeutic used in the clinical management of breast cancer, was unchanged with HMGCR knockdown (S2 Fig).

### Atorvastatin more potently suppresses proliferation of breast cancer cells *in vitro* than rosuvastatin

**Fig 2:**
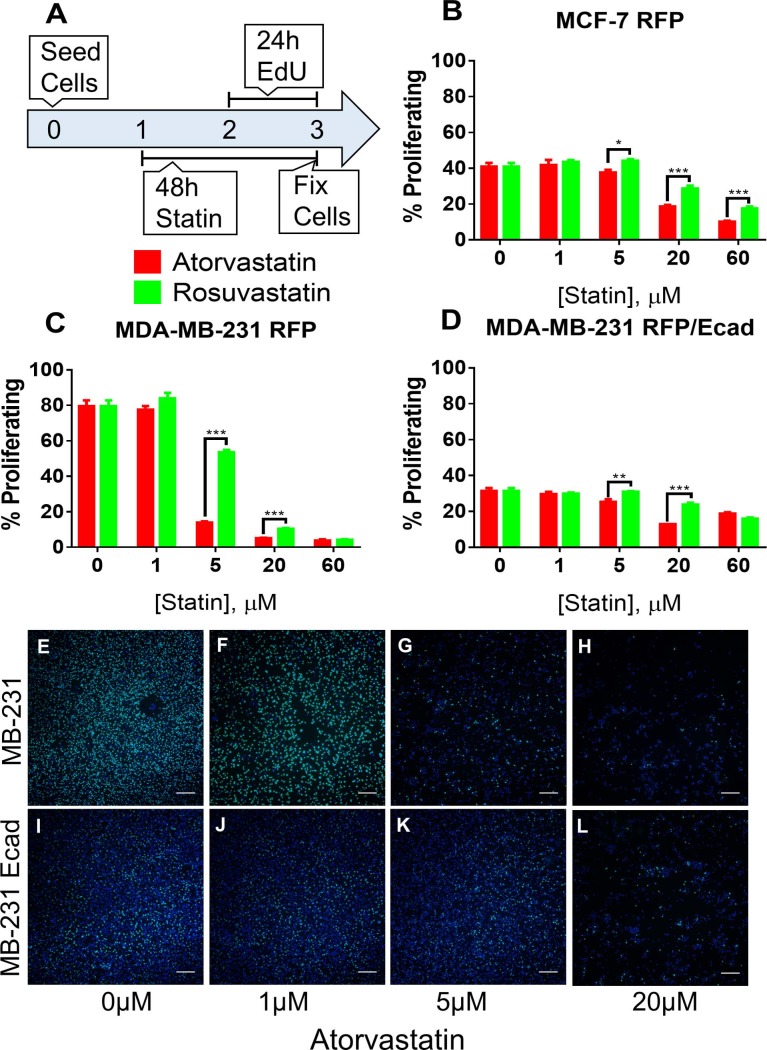
Atorvastatin decreases proliferation of breast cancer cells more potently than rosuvastatin. (A) Experimental schematic for assessing the proliferation of breast cancer cells under treatment with atorvastatin or rosuvastatin for 48 hours. (B) MCF-7 RFP, (C) MDA-MB-231 RFP, and (D) MDA-MB-231 RFP/Ecad were cultured with atorvastatin or rosuvastatin for 48 hours; during the final 24 hours the media included 10uM EdU. Cells were fixed, EdU was detected, and cells were counterstained with DAPI to label all nuclei. Cellular proliferation was quantified by determining the percentage of EdU positive cells (green, all nuclei are blue—DAPI). (E-H) MDA-MB-231 RFP and (I-L) MDA-MB-231 RFP/Ecad cells treated with (E,I) 0μM, (F,J) 1μM, (G,K) 5μM, or (H,L) 20μM atorvastatin demonstrate both E-cadherin mediated growth suppression and atorvastatin resistance. All data are representative of at least three independent experiments. * P < 0.05, ** P < 0.01, *** P < 0.001.

Since we observed atorvastatin was more effective at suppressing cell growth than rosuvastatin, we wanted to determine if this growth suppression was due to a decrease in cellular proliferation. Focusing on the breast cancer cell lines, we treated MCF-7 RFP, MDA-MB-231 RFP, or MDA-MB-231 RFP/Ecad with atorvastatin for 48 hours, incorporating EdU (a DNA analog) in the last 24 hours of treatment to quantify proliferation (Fig 2A). We observed a dose dependent decrease in the percentage of proliferating cells exposed to both atorvastatin and rosuvastatin in MCF-7 RFP (Fig 2B), MDA-MB-231 RFP (Fig 2C), and MDA-MB-231 RFP/Ecad (Fig 2D). Concurrent with our growth curve data, we found that atorvastatin was more effective at suppressing cell proliferation than rosuvastatin at the same treatment dosage.

We previously and herein demonstrate a higher resistance of membrane E-cadherin expressing MDA-MB-231 cancer cells to atorvastatin than their E-cadherin negative counterpart. When we examined the percentage of cells that had proliferated after 24 hours in the absence of statin treatment, we observed that the E-cadherin expressing MDA-MB-231 cells were roughly 50% less proliferative than the cells lacking E-cadherin (Fig 2E–2L). Moreover, similarly to the growth inhibition data, E-cadherin expressing MDA-MB-231 cells demonstrate less of a reduction in proliferating cells with statin treatment.

### Atorvastatin treatment induces cellular death in statin sensitive MDA-MB-231 cells but not statin resistant MCF-7 cells

**Fig 3:**
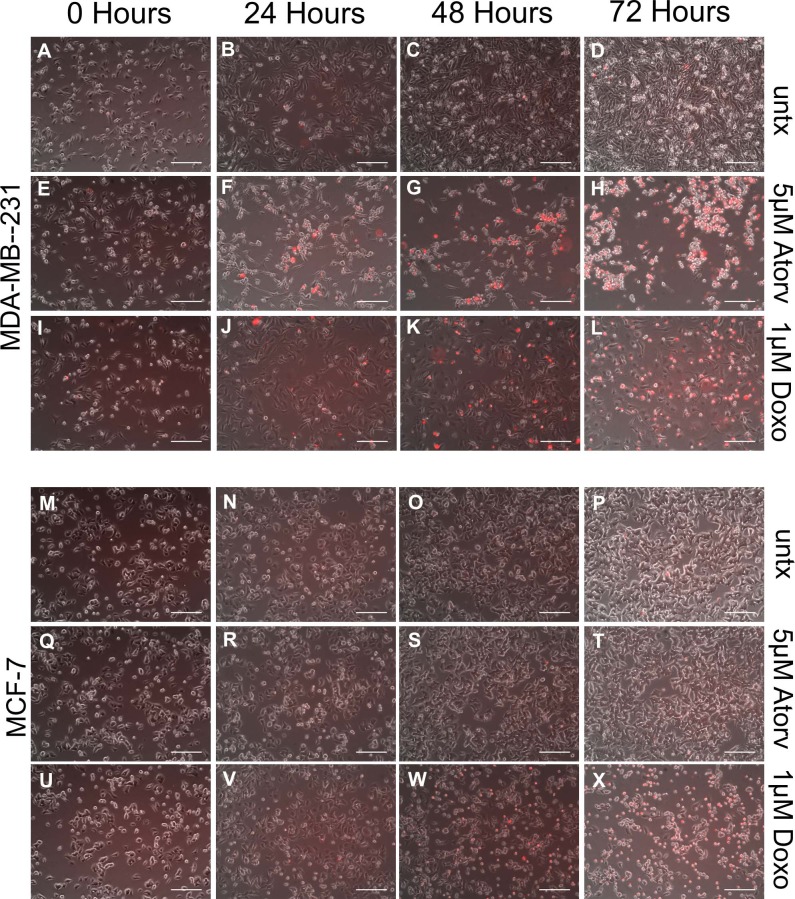
Atorvastatin decreases survival of sensitive but not resistant breast cancer cells. (A-L) MDA-MB-231 or (M-X) MCF-7 breast cancer cells were treated with 0.01% DMSO (untx, A-D and M-P), 5μM atorvastatin (5uM Atorv, E-H and Q-T), or 1μM Doxorubicin (1uM Doxo, I-L and U-X) for 0 hours, 24 hours, 48 hours, or 72 hours in the presence of 1μg/mL propidium iodide. Cells were imaged using an inverted microscope to detect propidium iodide (red) and look at cell morphology and density using phase contrast microscopy. Scale bar = 200μm. All data are representative of at least three independent experiments.

Given the anti-proliferative effects we observed of atorvastatin on breast cancer cells, we next wanted to determine whether atorvastatin also impacts individual cell survival. We treated statin sensitive MDA-MB-231 cells and statin resistant MCF-7 cells with 5μM atorvastatin for 72 hours and assayed cell death by including propidium iodide in the culture medium. We took phase contrast and fluorescent images every 24 hours and found that atorvastatin reduced the survival of MDA-MB-231 cells starting 24 hours after treatment (Fig 3A–3H). In contrast, the survival of MCF-7 cells was unaffected by atorvastatin even at 72 hours of treatment (Fig 3M–3T). The survival of both cell lines was decreased with 1μM of doxorubicin (Fig 3I–3L and 3U–3X). These data suggest that cell viability is reduced with atorvastatin treatment in cell lines that are susceptible to its growth inhibitory (Fig 1) and anti-proliferative (Fig 2) effects. Importantly, these data are an independent finding from the proliferative data shown in Fig 2, as the latter were normalized to the total adherent cell number which omits dead cells.

### Atorvastatin treatment decreases the proportion of membrane-tethered Ras in statin sensitive cells

**Fig 4:**
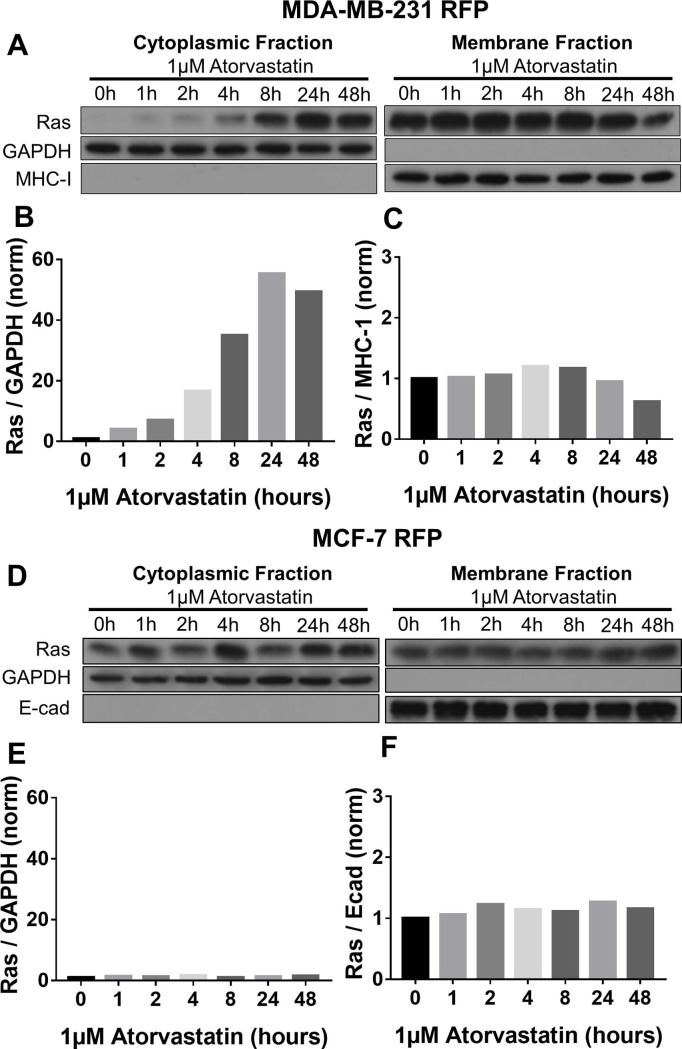
Atorvastatin treatment decreases membrane-bound Ras in statin sensitive MDA-MB-231 but not statin resistant MCF-7. (A-C) MDA-MB-231 RFP and (D-F) MCF-7 RFP cells were treated with atorvastatin for 0, 1, 2, 4, 8, 24, or 48 hours and protein was collected in cytoplasmic and membrane fractions. (A,B) Cytoplasmic Ras increased in statin treated MDA-MB-231 RFP cells over the course of 48 hours whereas (A,C) membrane Ras decreased. (D,E) Cytoplasmic and (D,F) membrane Ras were unchanged by atorvastatin treatment in MCF-7 RFP cells. All data are representative of at least three independent experiments.

To ascertain the mechanism by which atorvastatin treatment decreases both proliferation and survival, we queried whether a product of HMG-CoA reductase, which can be rescued by mevalonate bypassing the blockade of HMG-CoA reductase, was involved. These products are involved with protein lipidation to redirect otherwise soluble proteins to the cellular membranes. Thus, we investigated effects on the canonical intermediary pathways that contribute to both proliferation and survival in cancer cells, those thru MEK-Erk and PI3-kinase-Akt. Both of these can be activated downstream of Ras signaling, and Ras activation requires juxtamembrane positioning accomplished by protein prenylation. As such, we investigated Ras prenylation in statin sensitive MDA-MB-231 RFP and statin resistant MCF-7 RFP cells when treated with 1μM atorvastatin to determine if there is altered Ras signaling or downstream elements. We observe that statin sensitive MDA-MB-231 RFP cells show a 50-fold increase in cytoplasmic Ras and 50% decrease in membrane-bound Ras over 72 hours of treatment (Figs 4A–4C and S3). This is consistent with most of Ras being membrane-associated and statins not completely eliminating the production of geranyl-geranylphosphate. After prolonged statin treatment, we see degradation of cytoplasmic Ras, as has been previously reported in the literature [32]. Moreover, we found that EGF-stimulated activation of Ras was impaired with atorvastatin pre-treatment in MDA-MB-231 RFP cells (S4 Fig). In contrast, Ras localization is unchanged with atorvastatin treatment in statin resistant MCF-7 RFP cells (Fig 4D–4F). These data suggest that atorvastatin treatment in statin sensitive cells decreases the proportion of signaling competent Ras.

### Atorvastatin treatment blunts EGF-stimulated phosphorylation of Erk and Akt in a sensitivity-dependent manner

**Fig 5:**
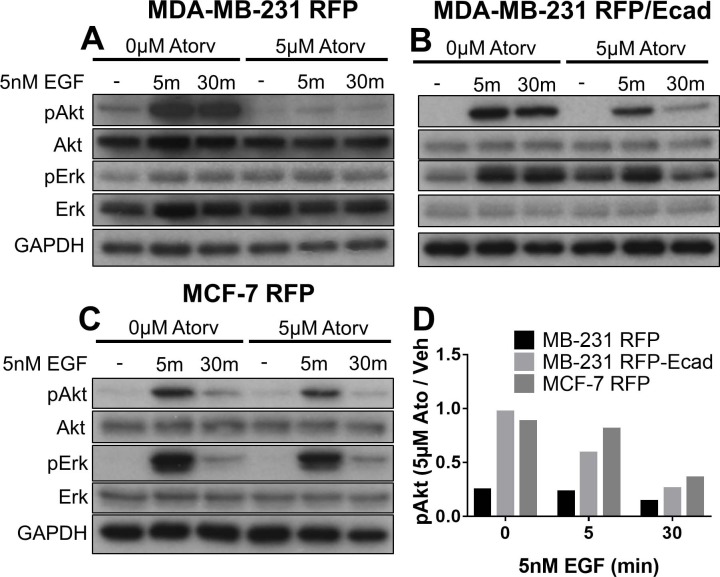
Atorvastatin sensitivity correlates with blunted Akt phosphorylation in response to EGF. (A) MDA-MB-231 RFP, (B) MDA-MB-231 RFP/Ecad, and (C) MCF-7 RFP cells were treated with 5μM atorvastatin for 24 hours and then stimulated with 5nM EGF for 5 or 30 minutes. (D) Akt phosphorylation fold change, defined as the density of pAkt under atorvastatin pretreatment divided by the density of pAkt under vehicle treatment, was quantified for 0, 5, or 30 minutes of 5nM EGF stimulation. All data are representative of at least three independent experiments.

EGF is a growth factor that signals proliferation and migration in breast cancer, and is present as an autocrine stimulatory factor in most all aggressive mammary carcinomas. Our lab has previously shown that EGF stimulation of dormant breast cancer cells can drive outgrowth from dormancy in an ex vivo microphysiological system model for breast cancer metastasis to the liver [33–35]. Given the importance of EGF in promoting breast cancer growth and metastasis, we wanted to determine whether atorvastatin pre-treatment could influence the phosphorylation of Akt and Erk in breast cancer cells. We pre-treated MCF-7 RFP, MDA-MB-231 RFP, or MDA-MB-231 RFP/Ecad with 5μM atorvastatin for 24 hours then stimulated cells with 5nM EGF for either 5 or 30 minutes. We found that atorvastatin pre-treatment was able to blunt EGF-mediated increases in Akt phosphorylation in all three cell lines and Erk-mediated phosphorylation in MDA-MB-231 RFP/Ecad and MCF-7 RFP cell lines (Fig 5A–5C). MDA-MB-231 RFP has high levels of tonic Erk phosphorylation due to autocrine EGF signaling [2].

We next quantified the blunted EGF response by comparing the vehicle pre-treatment to atorvastatin pre-treatment at each time point for EGF stimulation. With no EGF stimulation, we found that atorvastatin decreased the basal phosphorylation of Akt in statin sensitive MDA-MB-231 RFP cells but not in the more resistant MDA-MB-231 RFP/Ecad or MCF-7 RFP cells. With 5 or 30 minutes of EGF stimulation, we observed that the degree to which atorvastatin pre-treatment decreased EGF-stimulated Akt phosphorylation directly correlated with the sensitivity of these cell lines to atorvastatin-mediated growth inhibition (Figs 1 and 5D). These data suggest that the susceptibility to statin-mediated growth inhibition involves inhibition of Akt signaling and were consistent with the decrement in Ras localization to the membrane.

### Inhibition of Akt but not Erk signaling is synergistic with atorvastatin-mediated growth suppression

To explore what may potentiate the anti-proliferative effects of atorvastatin, we investigated the two effected pathways: the PI3K-Akt and MAP kinase signaling networks as both are downstream from Ras signaling and at least partially inhibited by statins. It has previously been shown that both pathways play important roles in breast cancer cell growth and migration [36,37]. To probe these signaling pathways, we employed a pan-PI3K inhibitor (LY294002) and a Mek1/2 inhibitor (PD98059), which inhibit their phosphorylation of Akt and Erk1/2 respectively. As crosstalk between these two pathways has been previously reported, we first determined the effect of each inhibitor on the opposing pathway. While the PI3K inhibitor increased Erk phosphorylation in a dose-dependent manner, we saw no effect of the Mek1/2 inhibitor on Akt phosphorylation (S5 Fig).

**Fig 6:**
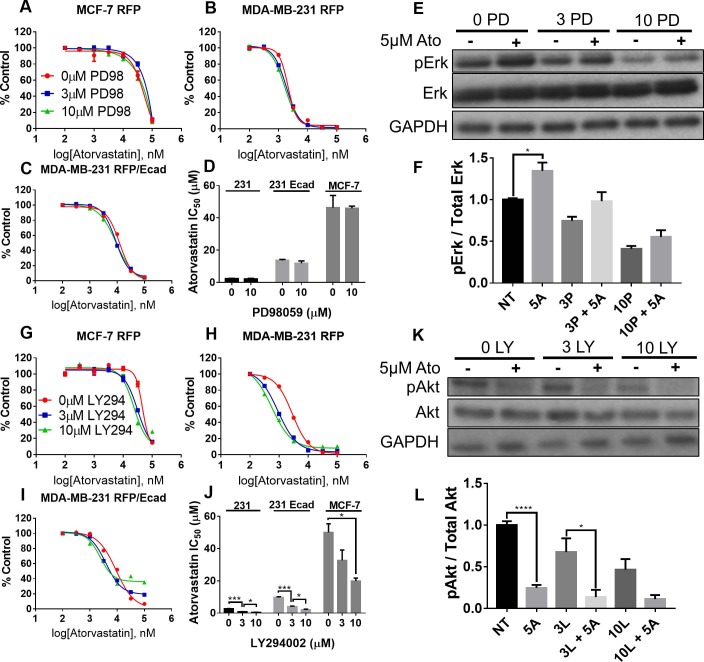
Inhibition of Akt but not Erk signaling is synergistic with atorvastatin. (A) MCF-7 RFP, (B) MDA-MB-231 RFP, and (C) MDA-MB-231 RFP/Ecad cells were cultured with atorvastatin and either 0μM, 3μM, or 10μM PD98059 for 72 hours. (D) IC_50_ values for atorvastatin susceptibility were extrapolated from sigmoid curve fits to the dose response data. (E) MDA-MB-231 RFP cells were treated with 0μM, 3μM, or 10μM PD98059 with or without 5μM atorvastatin for 24 hours and probed by western blot and (F) quantified by densitometry. (G) MCF-7 RFP, (H) MDA-MB-231 RFP, and (I) MDA-MB-231 RFP/Ecad cells were cultured with atorvastatin and either 0μM, 3μM, or 10μM LY294002 for 72 hours. (J) IC_50_ values for atorvastatin susceptibility were extrapolated from sigmoid curve fits to the dose response data. (K) MDA-MB-231 RFP cells were treated with 0μM, 3μM, or 10μM LY294002 with or without 5μM atorvastatin for 24 hours and probed by western blot and (L) quantified by densitometry. * P < 0.05, *** P < 0.001, and **** P < 0.0001. All data are representative of at least three independent experiments.

To probe the effects of these two inhibitors on influencing the susceptibility of breast cancer cells to atorvastatin, we co-treated MCF-7 RFP, MDA-MB-231 RFP, or MDA-MB-231 RFP/Ecad with atorvastatin and either PD98059 or LY294002 for 72 hours. With PD98059 and atorvastatin co-treatment, we observed no change in the atorvastatin IC_50_ to cell growth inhibition (Fig 6A–6D). Additionally, we observed an increase in Erk phosphorylation with 5μM atorvastatin treatment in statin sensitive MDA-MB-231 RFP cells at each dose of PD98059 used (Fig 6E and 6F). In contrast, we observed a dose-dependent potentiation of atorvastatin with LY294002 co-treatment (Fig 6G–6J). When probing Akt phosphorylation after atorvastatin treatment, we found that 5μM atorvastatin significantly decreased basal Akt phosphorylation in statin sensitive MDA-MB-231 RFP cells which was further decreased with LY294002 treatment (Fig 6K and 6L).

These data suggest the synergy between atorvastatin and PI3K inhibition may be due to suppression of Akt signaling. Moreover, since we observed an increase in Erk phosphorylation with our PI3K inhibitor, it is likely that the atorvastatin-mediated decrease in Akt phosphorylation is also causing the increase in Erk phosphorylation we observe in atorvastatin treated cells.

## Discussion

The metastatic cascade begins with an epithelial to mesenchymal transition (EMT), followed by select cancer cells detaching from the primary tumor. These cells then invade through the basement membrane and intravasate into the vasculature. Cells that survive in the circulation reach distant sites and, upon arrival to their tissue target, extravasate into the parenchyma. They then undergo a mesenchymal to epithelial reverting transition (MErT) and integrate into the new microenvironment as micrometastases [2]. Following a period of dormancy, which can last years or even decades, micrometastases undergo a second EMT and outgrow to form clinically evident metastases [3]. Distant micro-metastases bear poor prognosis for cancer patients, with five-year survival rates ranging from 2–28%, depending on the location of the primary tumor [1]. Preventing dissemination or micrometastatic outgrowth would delay this mortal stage in cancer progression. Unfortunately, by the time the primary tumor has been found, many tumor cells may have already disseminated to distant sites and established dormant micrometastases [4]. The clinical challenge in targeting dormant micrometastases is that their quiescent cells exhibit chemoresistance to many available standard therapies, which mostly target dividing cells [5]. Therefore, there is a great need for alternative therapies that either prevent metastasis initiation or suppress micrometastatic emergence.

Statins have been implicated in decreasing death from breast cancer in a manner consistent with an effect on metastases and not primary carcinogenesis. To understand this segregations, we previously demonstrated that statins are candidate drugs that selectively target cells undergoing EMT. We determined membrane E-cadherin to be a resistance marker for statin-mediated growth inhibition and demonstrated that the exogenous expression of membrane E-cadherin in a statin-sensitive cell line was sufficient to decrease statin potency [14]. In this study, we demonstrate that statins’ pharmacologic properties guide their propensity for cancer growth inhibition. We found that the hydrophilic rosuvastatin was less effective at suppressing cancer cell growth than lipophilic atorvastatin (Fig 1), even though the former has higher affinity for HMGCR (5.4nM vs. 8.2nM) [24]. It is important to note that the statin concentrations used in these studies are difficult to correlate to human dosing, but experiments in mice suggest that anti-tumor effects would be seen with doses used for clinical lipid lowering therapy [38–40]. Another hydrophilic statin, pravastatin, which has a low affinity for HMGCR (44.1nM) [24], was found to have no influence on cancer cell growth up to 100μM, even in sensitive cell lines. Yet, even this relatively ineffective statin could be potentiated by knockdown of HMGCR (Figs 1 and S2). The inefficacy of pravastatin has been previously reported [13] and is likely due to a combination of low affinity for HMGCR and hydrophilicity. Whereas lipophilic statins easily diffuse across the membrane, hydrophilic statins rely more on active transport. The four major statin transporters in the liver are SLCO1B1, 1B3, 1A2, 2B1 [41]. While statin transport is handled by all of these carriers, the major part of Atorvastatin handling is accomplished by SLCO1B1, which is why we focused on this transporter. Additionally, mutations in the SLCO1B1 gene are particularly known to predispose to statin-induced myopathy [42]. Indeed, exogenous expression of SLCO1B1 has been found to increase pravastatin uptake [43]. Unfortunately we were not able to stably express SLCO1B1 transporters in our cells to directly demonstrate this causality. In sum, our data suggest that the low efficacy of hydrophilic and low affinity statins is due to decreased HMGCR inhibition. This underscores the importance of the mevalonate pathway for cancer cell growth.

In this work, we suggest statins suppress tumor cell growth by two main mechanisms: 1) they decrease tumor cell proliferation (Fig 2), and 2) they decrease tumor cell survival (Fig 3). These two findings are significant and distinct because they suggest a bi-functionality of statins as anti-tumor agents, at least in vitro. Other authors have reported direct induction of apoptosis and cell death in tumor cells after statin treatment, in cancer cell lines derived from breast [44], lung [45], and prostate [46] as well as decreases in cell number or viability [15], with only few reporting decreased proliferation of statin treated cancer cells [47] by directly quantifying cycling cells. The majority of studies in the literature use methods that do not distinguish between cell viability and growth, such as MTT and ATP based assays. We show a decrease in proliferating percentage of adherent breast cancer cells (those that have survived the anti-survival effects of statin therapy) which demonstrates direct effects on cell proliferation (Fig 2). Moreover, the uptake of propidium iodide into statin sensitive MDA-MB-231 cells but not statin resistant MCF-7 cells starting at 24 hours after treatment demonstrates direct decreases in cell survival with statin therapy (Fig 3). We thus show in vitro that statins act to both decrease survival and proliferation in breast cancer cells.

It has been postulated that part of the anti-tumor effect of statins is caused by reduction in the prenylation, and thus localization, of signaling proteins such as Ras and the Rho family of proteins [19]. Decreases in membrane anchoring of H-Ras have been shown in breast cancer cells treated with statins [48]. In this study, we demonstrate that the localization of Ras is dependent on the statin sensitivity of the cells undergoing treatment. While the statin sensitive MDA-MB-231 RFP cells demonstrate a shift from membrane to cytoplasmic Ras localization over the course of 72 hours of statin treatment, the same increases are not seen in statin resistant MCF-7 RFP cells (Figs 4 and S3). Importantly, the decrease in membrane localization of Ras correlated with a decrease in EGF-mediated Ras activation (S4 Fig). Thus, the influence of statins on the prenylation status of Ras, which affects its subcellular localization, correlates with the sensitivity of the cells to statin-mediated growth suppression.

To examine intracellular networks that may be responsible for the reduction in cell survival and proliferation, we independently inhibited the MAPK and PI3K-Akt signaling axes. We found that reduction of Akt signaling through PI3K inhibition significantly potentiated the growth suppression of atorvastatin whereas reduction of Erk signaling through Mek1/2 inhibition did not affect atorvastatin efficacy (Fig 6). Western blotting demonstrated a significant reduction of Akt phosphorylation in atorvastatin treated MDA-MB-231 RFP cells whereas Erk phosphorylation was increased with atorvastatin treatment (Fig 6). Or it could be simply that the inhibition of the MEK/Erk pathway was redundant with the decrement of Ras signaling. Previous studies have demonstrated crosstalk between the PI3K-Akt and MAPK signaling pathways at the level of Akt and Raf [49]. Indeed, we demonstrate PI3K inhibition increases Erk phosphorylation whereas Mek1/2 inhibition does not affect Akt phosphorylation (S5 Fig). These findings suggest that the reduction in Akt phosphorylation observed with statin treatment may be the source of the increase in Erk phosphorylation. Others have demonstrated decreases in Akt phosphorylation with statin treatment in prostate [50] and breast [51] cancer cell lines.

As stated previously, micrometastases often remain in a state of dormancy for years or decades before emerging as clinically evident metastases. Our lab has previously demonstrated that dormant micrometastases can be stimulated to outgrowth in an ex vivo microphysiological system model for breast cancer metastasis to the liver by using a combined stimulus of EGF and LPS [33]. To determine whether atorvastatin would influence the EGF responsiveness of our breast cancer cell lines, we treated MCF-7 RFP, MDA-MB-231 RFP, and MDA-MB-231 RFP/Ecad with atorvastatin for 24 hours and then stimulated with 5nM EGF for 5 or 30 minutes. We found that all three cell lines demonstrated increases in Akt and Erk phosphorylation with EGF stimulation and that atorvastatin pretreatment could block these increases in a manner that correlated with their sensitivity to atorvastatin-mediated growth inhibition (Fig 5). The decrease in Akt phosphorylation seen after 30 minutes of EGF treatment is most likely secondary to receptor internalization and degradation due to the high abundance of soluble ligands [52,53]. These data suggest that atorvastatin may decrease growth-factor stimulated growth of breast cancer cells. Moreover, the E-cadherin mediated resistance to atorvastatin suppression of Akt stimulation by EGF suggests that epithelial micrometastases that undergo a secondary EMT and outgrow will be more selectively suppressed by statin treatment than those that remain in dormancy. As atorvastatin effects are most noted when E-cadherin is absent from the plasma membrane [14], the cells that are epithelial and dormant at micrometastases would likely be relatively unaffected. However, when these cancer cells undergo a secondary EMT that limits E-cadherin presentation during metastatic outgrowth, they would then be susceptible to statin suppression.

The differing potencies of statin drugs have important clinical implications. Our data suggest lipophilic statins may be more effective at suppressing micrometastatic outgrowth because they have increased uptake into cancer cells. For cancer patients already receiving statins for other conditions, such as hypercholesterolemia, changing to a more potent anti-cancer statin, such as atorvastatin, may provide a mortality benefit without adversely affecting their primary indication for statin therapy.

## Supporting information

S1 Table Pharmacologic properties of statins. The IC50 values for inhibition of HMGCR in cell-free binding assays [24] and partition coefficients [31] are reported for atorvastatin, simvastatin, rosuvastatin, and pravastatin. The lower the IC50 value, the more potent the statin. The lower the partition coefficient, the more hydrophilic the statin.
(TIF)

S1 Fig Simvastatin exhibits similar growth suppressive potency to atorvastatin. (A) MCF-7 RFP and (B) MDA-MB-231 RFP cells were cultured with atorvastatin or simvastatin for 72 hours and cell number was determined by crystal violet staining. (C) MCF-7 RFP and (D) MDA-MB-231 RFP cells were cultured with simvastatin lactone (un-activated) or simvastatin acid (activated) for 72 hours and cell number was determined by crystal violet staining. All data are representative of at least three independent experiments.
(TIF)

S2 Fig HMGCR knockdown decreases cell growth and potentates statin therapy. HMGCR was knocked down by siRNA treatment in MDA-MB-231 cells and cells were subsequently treated with (A,D) atorvastatin, (B,E) doxorubicin, or (C,F) pravastatin for 72 hours. (A-C) Data were normalized to the non-coding RNA control and then (D-F) further normalized to the lowest dose of drug used. (G) IC_50_ values for atorvastatin (Atorv), doxorubicin (Dox), and pravastatin (Prav) were calculated based on sigmoid curve fits to the dose response data. (H) HMGCR immunoblotting 24, 48, and 72 hours after siRNA knockdown with (I) quantification by densitometry. * P < 0.05. All data are representative of at least three independent experiments.
(TIF)

S3 Fig Ras localization is altered in MDA-MB-231 RFP cells over 72 hours of atorvastatin treatment. (A) MDA-MB-231 RFP cells were treated with 1μM atorvastatin for 0, 24, 48, or 72 hours and protein was collected in cytoplasmic and membrane fractions and probed by western blot. (B) Cytoplasmic Ras and (C) membrane Ras were quantified by densitometry. All data are representative of at least three independent experiments.
(TIF)

S4 Fig Atorvastatin pre-treatment reduces EGF-stimulated Ras activation. MDA-MB-231 RFP cells were treated with or without 1μM atorvastatin for 48 hours and then cells were stimulated with 5nM EGF for 5 minutes. Activated Ras (Ras-GTP) was isolated from cell lysates, (A,B) probed by western blot, and (C) quantified by densitometry of the faster mobility fraction. Atorv = Atorvastatin, NT = No treatment, A = 1uM Atorvastatin for 48 hours, EGF = 5nM EGF for 5 minutes. Error bars represent the SEM. * P < 0.05. All data are representative of at least three independent experiments.
(TIF)

S5 Fig PI3K inhibition enhances Erk phosphorylation but Mek inhibition does not affect Akt phosphorylation. MDA-MB-231 RFP cells were treated with or without 5μM atorvastatin supplemented with (A) 0μM, 3μM, or 10μM LY294002 an inhibitor of PI3 kinase or (B) 0μM, 3μM, or 10μM PD98059 and inhibitor of MEK for 24 hours and (A) pErk and total Erk or (B) pAkt and total Akt were probed by western blot. Importantly, the distinction being made is with increasing doses of either LY294002 or PD98059 (comparing lanes 1, 3, and 5). The effect of atorvastatin treatment (comparing lanes 1 & 2, 3 & 4, and 5 & 6) on Akt and Erk phosphorylation is the same as shown in Fig 6. All data are representative of at least three independent experiments.
(TIF)

## References

[1] Siegel RL, Miller KD, Jemal A. Cancer statistics, 2016 CA Cancer J Clin [Internet]. 66(1):7–30. Available from: http://www.ncbi.nlm.nih.gov/pubmed/26742998 doi: 10.3322/caac.21332 26742998 10.3322/caac.21332

[2] Chao YL, Shepard CR, Wells A. Breast carcinoma cells re-express E-cadherin during mesenchymal to epithelial reverting transition. Mol Cancer [Internet]. 2010 7 7;9:179 Available from: http://www.ncbi.nlm.nih.gov/pubmed/20609236 doi: 10.1186/1476-4598-9-179 20609236 10.1186/1476-4598-9-179 PMC2907333

[3] Aguirre-Ghiso JA. Models, mechanisms and clinical evidence for cancer dormancy. Nat Rev Cancer [Internet]. 2007 11;7(11):834–46. Available from: http://www.ncbi.nlm.nih.gov/pubmed/17957189 doi: 10.1038/nrc2256 17957189 10.1038/nrc2256 PMC2519109

[4] Chambers AF, Groom AC, MacDonald IC. Dissemination and growth of cancer cells in metastatic sites. Nat Rev Cancer [Internet]. 2002 8;2(8):563–72. Available from: http://www.ncbi.nlm.nih.gov/pubmed/12154349 doi: 10.1038/nrc865 12154349 10.1038/nrc865

[5] Wells A, Griffith L, Wells JZ, Taylor DP. The Dormancy Dilemma: Quiescence versus Balanced Proliferation. Cancer Res [Internet]. 2013 7 1;73(13):3811–6. Available from: http://cancerres.aacrjournals.org/lookup/doi/10.1158/0008-5472.CAN-13-0356 doi: 10.1158/0008-5472.CAN-13-0356 23794703 10.1158/0008-5472.CAN-13-0356 PMC3702639

[6] Allison M. NCATS launches drug repurposing program. Nat Biotechnol [Internet]. 2012 7 10;30(7):571–2. Available from: http://www.nature.com/doifinder/10.1038/nbt0712-571a doi: 10.1038/nbt0712-571a 22781662 10.1038/nbt0712-571a

[7] Deftereos SN, Andronis C, Friedla EJ, Persidis A, Persidis A. Drug repurposing and adverse event prediction using high-throughput literature analysis. Wiley Interdiscip Rev Syst Biol Med [Internet]. 2011 5;3(3):323–34. Available from: http://doi.wiley.com/10.1002/wsbm.147 doi: 10.1002/wsbm.147 21416632 10.1002/wsbm.147

[8] ENDO A. A historical perspective on the discovery of statins. Proc Japan Acad Ser B [Internet]. 2010;86(5):484–93. Available from: http://joi.jlc.jst.go.jp/JST.JSTAGE/pjab/86.484?from=CrossRef 20467214 10.2183/pjab.86.484 PMC3108295

[9] Nielsen SF, Nordestgaard BG, Bojesen SE. Statin use and reduced cancer-related mortality. N Engl J Med [Internet]. 2012 11 8;367(19):1792–802. Available from: http://www.ncbi.nlm.nih.gov/pubmed/23134381 doi: 10.1056/NEJMoa1201735 23134381 10.1056/NEJMoa1201735

[10] Wang A, Aragaki AK, Tang JY, Kurian AW, Manson JE, Chlebowski RT, et al Statin use and all-cancer survival: prospective results from the Women’s Health Initiative. Br J Cancer [Internet]. 2016 6 28;115(1):129–35. Available from: http://www.ncbi.nlm.nih.gov/pubmed/27280630 doi: 10.1038/bjc.2016.149 27280630 10.1038/bjc.2016.149 PMC4931370

[11] Haukka J, Sankila R, Klaukka T, Lonnqvist J, Niskanen L, Tanskanen A, et al Incidence of cancer and statin usage-Record linkage study. Int J Cancer [Internet]. 2010 1 1;126(1):279–84. Available from: http://doi.wiley.com/10.1002/ijc.24536 doi: 10.1002/ijc.24536 19739258 10.1002/ijc.24536

[12] Shepherd J, Blauw GJ, Murphy MB, Bollen ELEM, Buckley BM, Cobbe SM, et al Pravastatin in elderly individuals at risk of vascular disease (PROSPER): a randomised controlled trial. Lancet (London, England) [Internet]. 2002 11 23;360(9346):1623–30. Available from: http://www.ncbi.nlm.nih.gov/pubmed/12457784 10.1016/s0140-6736(02)11600-x 12457784

[13] Menter DG, Ramsauer VP, Harirforoosh S, Chakraborty K, Yang P, Hsi L, et al Differential effects of pravastatin and simvastatin on the growth of tumor cells from different organ sites. PLoS One [Internet]. 2011;6(12):e28813 Available from: http://www.ncbi.nlm.nih.gov/pubmed/22216116 doi: 10.1371/journal.pone.0028813 22216116 10.1371/journal.pone.0028813 PMC3245236

[14] Warita K, Warita T, Beckwitt CH, Schurdak ME, Vazquez A, Wells A, et al Statin-induced mevalonate pathway inhibition attenuates the growth of mesenchymal-like cancer cells that lack functional E-cadherin mediated cell cohesion. Sci Rep [Internet]. 2014 12 23;4:7593 Available from: http://www.ncbi.nlm.nih.gov/pubmed/25534349 doi: 10.1038/srep07593 25534349 10.1038/srep07593 PMC4274516

[15] Campbell MJ, Esserman LJ, Zhou Y, Shoemaker M, Lobo M, Borman E, et al Breast cancer growth prevention by statins. Cancer Res [Internet]. 2006 9 1;66(17):8707–14. Available from: http://www.ncbi.nlm.nih.gov/pubmed/16951186 doi: 10.1158/0008-5472.CAN-05-4061 16951186 10.1158/0008-5472.CAN-05-4061

[16] Hoque A, Chen H, Xu X-c. Statin Induces Apoptosis and Cell Growth Arrest in Prostate Cancer Cells. Cancer Epidemiol Biomarkers Prev [Internet]. 2008 1 9;17(1):88–94. Available from: http://cebp.aacrjournals.org/cgi/doi/10.1158/1055-9965.EPI-07-0531 doi: 10.1158/1055-9965.EPI-07-0531 18199714 10.1158/1055-9965.EPI-07-0531

[17] Denoyelle C, Vasse M, Körner M, Mishal Z, Ganné F, Vannier JP, et al Cerivastatin, an inhibitor of HMG-CoA reductase, inhibits the signaling pathways involved in the invasiveness and metastatic properties of highly invasive breast cancer cell lines: an in vitro study. Carcinogenesis [Internet]. 2001 8;22(8):1139–48. Available from: http://www.ncbi.nlm.nih.gov/pubmed/11470741 11470741 10.1093/carcin/22.8.1139

[18] Gbelcová H, Lenícek M, Zelenka J, Knejzlík Z, Dvoráková G, Zadinová M, et al Differences in antitumor effects of various statins on human pancreatic cancer. Int J cancer [Internet]. 2008 3 15;122(6):1214–21. Available from: http://www.ncbi.nlm.nih.gov/pubmed/18027870 doi: 10.1002/ijc.23242 18027870 10.1002/ijc.23242

[19] Gazzerro P, Proto MC, Gangemi G, Malfitano AM, Ciaglia E, Pisanti S, et al Pharmacological actions of statins: a critical appraisal in the management of cancer. Pharmacol Rev [Internet]. 2012 1;64(1):102–46. Available from: http://www.ncbi.nlm.nih.gov/pubmed/22106090 doi: 10.1124/pr.111.004994 22106090 10.1124/pr.111.004994

[20] Gelb MH. Protein prenylation, et cetera: signal transduction in two dimensions. Science [Internet]. 1997 3 21;275(5307):1750–1. Available from: http://www.ncbi.nlm.nih.gov/pubmed/9122679 9122679 10.1126/science.275.5307.1750

[21] Stancu C, Sima A. Statins: mechanism of action and effects. J Cell Mol Med [Internet]. 5(4):378–87. Available from: http://www.ncbi.nlm.nih.gov/pubmed/12067471 12067471 10.1111/j.1582-4934.2001.tb00172.x PMC6740083

[22] Ahern TP, Pedersen L, Tarp M, Cronin-Fenton DP, Garne JP, Silliman RA, et al Statin prescriptions and breast cancer recurrence risk: a Danish nationwide prospective cohort study. J Natl Cancer Inst [Internet]. 2011 10 5;103(19):1461–8. Available from: http://www.ncbi.nlm.nih.gov/pubmed/21813413 doi: 10.1093/jnci/djr291 21813413 10.1093/jnci/djr291 PMC3186780

[23] Kwan ML, Habel LA, Flick ED, Quesenberry CP, Caan B. Post-diagnosis statin use and breast cancer recurrence in a prospective cohort study of early stage breast cancer survivors. Breast Cancer Res Treat [Internet]. 2008 6 3;109(3):573–9. Available from: http://link.springer.com/10.1007/s10549-007-9683-8 doi: 10.1007/s10549-007-9683-8 17674197 10.1007/s10549-007-9683-8 PMC3507509

[24] McKenney JM. Pharmacologic characteristics of statins. Clin Cardiol [Internet]. 2003 4;26(4 Suppl 3):III32–8. Available from: http://www.ncbi.nlm.nih.gov/pubmed/12708637 12708637 10.1002/clc.4960261507 PMC6654766

[25] Kim N-G, Koh E, Chen X, Gumbiner BM. E-cadherin mediates contact inhibition of proliferation through Hippo signaling-pathway components. Proc Natl Acad Sci U S A [Internet]. 2011 7 19;108(29):11930–5. Available from: http://www.ncbi.nlm.nih.gov/pubmed/21730131 doi: 10.1073/pnas.1103345108 21730131 10.1073/pnas.1103345108 PMC3141988

[26] DeBerardinis RJ, Lum JJ, Hatzivassiliou G, Thompson CB. The biology of cancer: metabolic reprogramming fuels cell growth and proliferation. Cell Metab [Internet]. 2008 1;7(1):11–20. Available from: http://www.ncbi.nlm.nih.gov/pubmed/18177721 doi: 10.1016/j.cmet.2007.10.002 18177721 10.1016/j.cmet.2007.10.002

[27] Manning BD, Toker A. AKT/PKB Signaling: Navigating the Network. Cell [Internet]. 2017 4;169(3):381–405. Available from: http://linkinghub.elsevier.com/retrieve/pii/S0092867417304130 doi: 10.1016/j.cell.2017.04.001 28431241 10.1016/j.cell.2017.04.001 PMC5546324

[28] Mendoza MC, Er EE, Blenis J. The Ras-ERK and PI3K-mTOR pathways: cross-talk and compensation. Trends Biochem Sci [Internet]. 2011 6;36(6):320–8. Available from: http://linkinghub.elsevier.com/retrieve/pii/S0968000411000508 doi: 10.1016/j.tibs.2011.03.006 21531565 10.1016/j.tibs.2011.03.006 PMC3112285

[29] Grille SJ, Bellacosa A, Upson J, Klein-Szanto AJ, van Roy F, Lee-Kwon W, et al The protein kinase Akt induces epithelial mesenchymal transition and promotes enhanced motility and invasiveness of squamous cell carcinoma lines. Cancer Res [Internet]. 2003 5 1;63(9):2172–8. Available from: http://www.ncbi.nlm.nih.gov/pubmed/12727836 12727836

[30] Ma B, Wheeler SE, Clark AM, Whaley DL, Yang M, Wells A. Liver protects metastatic prostate cancer from induced death by activating E-cadherin signaling. Hepatology [Internet]. 2016 11;64(5):1725–42. Available from: http://www.ncbi.nlm.nih.gov/pubmed/27482645 doi: 10.1002/hep.28755 27482645 10.1002/hep.28755 PMC5074910

[31] Wishart DS, Feunang YD, Guo AC, Lo EJ, Marcu A, Grant JR, et al DrugBank 5.0: a major update to the DrugBank database for 2018. Nucleic Acids Res [Internet]. 2017 11 8; Available from: http://academic.oup.com/nar/article/doi/10.1093/nar/gkx1037/4602867 10.1093/nar/gkx1037 PMC5753335 29126136

[32] Haklai R, Weisz MG, Elad G, Paz A, Marciano D, Egozi Y, et al Dislodgment and Accelerated Degradation of Ras †. Biochemistry [Internet]. 1998 2;37(5):1306–14. Available from: http://pubs.acs.org/doi/abs/10.1021/bi972032d doi: 10.1021/bi972032d 9477957 10.1021/bi972032d

[33] Wheeler SE, Clark a M, Taylor DP, Young CL, Pillai VC, Stolz DB, et al Spontaneous dormancy of metastatic breast cancer cells in an all human liver microphysiologic system. Br J Cancer [Internet]. 2014;111(12):2342–50. Available from: doi: 10.1038/bjc.2014.533 25314052 10.1038/bjc.2014.533 PMC4264444

[34] Clark AM, Wheeler SE, Taylor DP, Pillai VC, Young CL, Prantil-Baun R, et al A microphysiological system model of therapy for liver micrometastases. Exp Biol Med (Maywood) [Internet]. 2014;1535370214532596. Available from: http://ebm.sagepub.com/content/early/2014/05/09/1535370214532596.full 10.1177/1535370214532596 PMC4574864 24821820

[35] Clark AM, Wheeler SE, Young CL, Stockdale L, Shepard Neiman J, Zhao W, et al A liver microphysiological system of tumor cell dormancy and inflammatory responsiveness is affected by scaffold properties. Lab Chip [Internet]. 2017;17(1):156–68. Available from: http://xlink.rsc.org/?DOI=C6LC01171C 10.1039/c6lc01171c PMC5242229 27910972

[36] Santen RJ, Song RX, McPherson R, Kumar R, Adam L, Jeng M-H, et al The role of mitogen-activated protein (MAP) kinase in breast cancer. J Steroid Biochem Mol Biol [Internet]. 2002 2;80(2):239–56. Available from: http://www.ncbi.nlm.nih.gov/pubmed/11897507 11897507 10.1016/s0960-0760(01)00189-3

[37] Paplomata E, O’Regan R. The PI3K/AKT/mTOR pathway in breast cancer: targets, trials and biomarkers. Ther Adv Med Oncol [Internet]. 2014 7;6(4):154–66. Available from: http://journals.sagepub.com/doi/10.1177/1758834014530023 doi: 10.1177/1758834014530023 25057302 10.1177/1758834014530023 PMC4107712

[38] Campbell MJ, Esserman LJ, Zhou Y, Shoemaker M, Lobo M, Borman E, et al Breast Cancer Growth Prevention by Statins. Cancer Res [Internet]. 2006 9 1;66(17):8707–14. Available from: http://cancerres.aacrjournals.org/lookup/doi/10.1158/0008-5472.CAN-05-4061 doi: 10.1158/0008-5472.CAN-05-4061 16951186 10.1158/0008-5472.CAN-05-4061

[39] Shibata M-A, Ito Y, Morimoto J, Otsuki Y. Lovastatin inhibits tumor growth and lung metastasis in mouse mammary carcinoma model: a p53-independent mitochondrial-mediated apoptotic mechanism. Carcinogenesis [Internet]. 2004 10 3;25(10):1887–98. Available from: https://academic.oup.com/carcin/article-lookup/doi/10.1093/carcin/bgh201 doi: 10.1093/carcin/bgh201 15180944 10.1093/carcin/bgh201

[40] Stone NJ, Robinson JG, Lichtenstein AH, Bairey Merz CN, Blum CB, Eckel RH, et al 2013 ACC/AHA Guideline on the Treatment of Blood Cholesterol to Reduce Atherosclerotic Cardiovascular Risk in Adults. J Am Coll Cardiol [Internet]. 2014 7;63(25):2889–934. Available from: http://linkinghub.elsevier.com/retrieve/pii/S0735109713060282 24239923 10.1016/j.jacc.2013.11.002

[41] Kalliokoski A, Niemi M. Impact of OATP transporters on pharmacokinetics. Br J Pharmacol [Internet]. 2009 10;158(3):693–705. Available from: http://www.ncbi.nlm.nih.gov/pubmed/19785645 doi: 10.1111/j.1476-5381.2009.00430.x 19785645 10.1111/j.1476-5381.2009.00430.x PMC2765590

[42] Stewart A. SLCO1B1 Polymorphisms and Statin-Induced Myopathy. PLoS Curr [Internet]. 2013; Available from: http://currents.plos.org/genomictests/?p=21697 10.1371/currents.eogt.d21e7f0c58463571bb0d9d3a19b82203 PMC3871416 24459608

[43] Zhang X, Scialis RJ, Feng B, Leach K. Detection of statin cytotoxicity is increased in cells expressing the OATP1B1 transporter. Toxicol Sci [Internet]. 2013 7;134(1):73–82. Available from: http://www.ncbi.nlm.nih.gov/pubmed/23564645 doi: 10.1093/toxsci/kft085 23564645 10.1093/toxsci/kft085

[44] Gopalan A, Yu W, Sanders BG, Kline K. Simvastatin inhibition of mevalonate pathway induces apoptosis in human breast cancer cells via activation of JNK/CHOP/DR5 signaling pathway. Cancer Lett [Internet]. 2013 2 1;329(1):9–16. Available from: http://www.ncbi.nlm.nih.gov/pubmed/22960596 doi: 10.1016/j.canlet.2012.08.031 22960596 10.1016/j.canlet.2012.08.031

[45] Pelaia G, Gallelli L, Renda T, Fratto D, Falcone D, Caraglia M, et al Effects of statins and farnesyl transferase inhibitors on ERK phosphorylation, apoptosis and cell viability in non-small lung cancer cells. Cell Prolif [Internet]. 2012 12;45(6):557–65. Available from: http://www.ncbi.nlm.nih.gov/pubmed/23045963 doi: 10.1111/j.1365-2184.2012.00846.x 23045963 10.1111/j.1365-2184.2012.00846.x PMC6496308

[46] Ben Sahra I, Laurent K, Giuliano S, Larbret F, Ponzio G, Gounon P, et al Targeting cancer cell metabolism: the combination of metformin and 2-deoxyglucose induces p53-dependent apoptosis in prostate cancer cells. Cancer Res [Internet]. 2010 3 15;70(6):2465–75. Available from: http://www.ncbi.nlm.nih.gov/pubmed/20215500 doi: 10.1158/0008-5472.CAN-09-2782 20215500 10.1158/0008-5472.CAN-09-2782

[47] Ogunwobi OO, Beales ILP. Statins inhibit proliferation and induce apoptosis in Barrett’s esophageal adenocarcinoma cells. Am J Gastroenterol [Internet]. 2008 4;103(4):825–37. Available from: http://www.ncbi.nlm.nih.gov/pubmed/18371146 doi: 10.1111/j.1572-0241.2007.01773.x 18371146 10.1111/j.1572-0241.2007.01773.x

[48] Kang S, Kim E-S, Moon A. Simvastatin and lovastatin inhibit breast cell invasion induced by H-Ras. Oncol Rep [Internet]. 2009 5;21(5):1317–22. Available from: http://www.ncbi.nlm.nih.gov/pubmed/19360310 19360310 10.3892/or_00000357

[49] Moelling K, Schad K, Bosse M, Zimmermann S, Schweneker M. Regulation of Raf-Akt Cross-talk. J Biol Chem [Internet]. 2002 8 23;277(34):31099–106. Available from: http://www.ncbi.nlm.nih.gov/pubmed/12048182 doi: 10.1074/jbc.M111974200 12048182 10.1074/jbc.M111974200

[50] Kochuparambil ST, Al-Husein B, Goc A, Soliman S, Somanath PR. Anticancer efficacy of simvastatin on prostate cancer cells and tumor xenografts is associated with inhibition of Akt and reduced prostate-specific antigen expression. J Pharmacol Exp Ther [Internet]. 2011 2;336(2):496–505. Available from: http://www.ncbi.nlm.nih.gov/pubmed/21059805 doi: 10.1124/jpet.110.174870 21059805 10.1124/jpet.110.174870

[51] Wang T, Seah S, Loh X, Chan C-W, Hartman M, Goh B-C, et al Simvastatin-induced breast cancer cell death and deactivation of PI3K/Akt and MAPK/ERK signalling are reversed by metabolic products of the mevalonate pathway. Oncotarget [Internet]. 2016 1 19;7(3):2532–44. Available from: http://www.ncbi.nlm.nih.gov/pubmed/26565813 doi: 10.18632/oncotarget.6304 26565813 10.18632/oncotarget.6304 PMC4823053

[52] Reddy CC, Wells A, Lauffenburger DA. Receptor-mediated effects on ligand availability influence relative mitogenic potencies of epidermal growth factor and transforming growth factor α. J Cell Physiol [Internet]. 1996 3;166(3):512–22. Available from: http://doi.wiley.com/10.1002/%28SICI%291097-4652%28199603%29166%3A3%3C512%3A%3AAID-JCP6%3E3.0.CO%3B2-S doi: 10.1002/(SICI)1097-4652(199603)166:3<512::AID-JCP6>3.0.CO;2-S 8600155 10.1002/(SICI)1097-4652(199603)166:3<512::AID-JCP6>3.0.CO;2-S

[53] Reddy CC, Wells A, Lauffenburger DA. Alteration of the Proliferative Response of Fibroblasts Expressing Internalization-Deficient Epidermal Growth Factor (EGF) receptors Is Altered via Differential EGF Depletion Effects. Biotechnol Prog [Internet]. 1994 7;10(4):377–84. Available from: http://doi.wiley.com/10.1021/bp00028a006 doi: 10.1021/bp00028a006 7765094 10.1021/bp00028a006

