# Supplementary figures and images for: Lipophilic statins limit cancer cell growth and survival, via involvement of Akt signaling
Source: PLoS One. 2018 May 15;13(5):e0197422. doi: 10.1371/journal.pone.0197422 (PMC5953490; doi:10.1371/journal.pone.0197422)

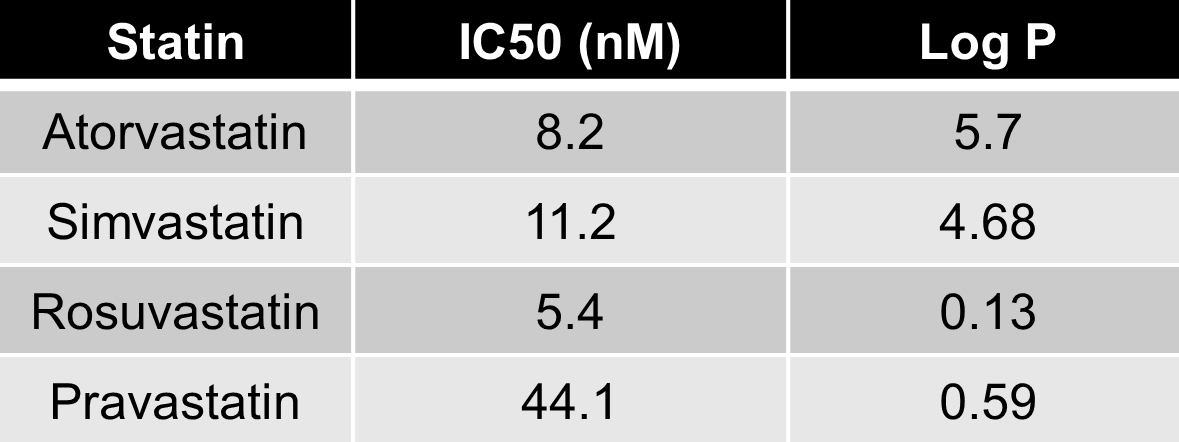

Supplement: S1 Table — The IC50 values for inhibition of HMGCR in cell-free binding assays [24] and partition coefficients [31] are reported for atorvastatin, simvastatin, rosuvastatin, and pravastatin. The lower the IC50 value, the more potent the statin. The lower the partition coefficient, the more hydrophilic the statin. (TIF) [file pone.0197422.s001.TIF]

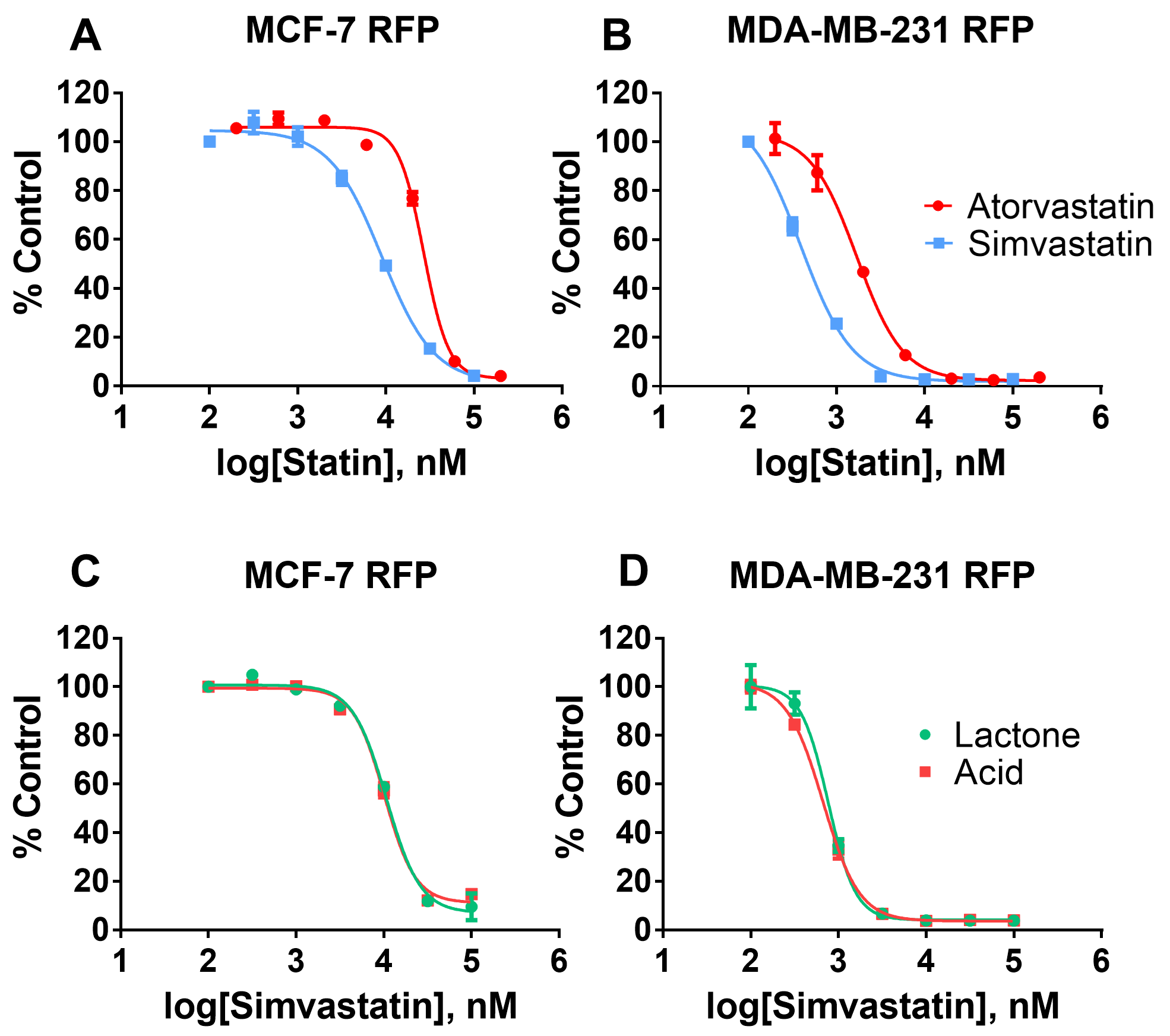

Supplement: S1 Fig — (A) MCF-7 RFP and (B) MDA-MB-231 RFP cells were cultured with atorvastatin or simvastatin for 72 hours and cell number was determined by crystal violet staining. (C) MCF-7 RFP and (D) MDA-MB-231 RFP cells were cultured with simvastatin lactone (un-activated) or simvastatin acid (activated) for 72 hours and cell number was determined by crystal violet staining. All data are representative of at least three independent experiments. (TIF) [file pone.0197422.s002.TIF]

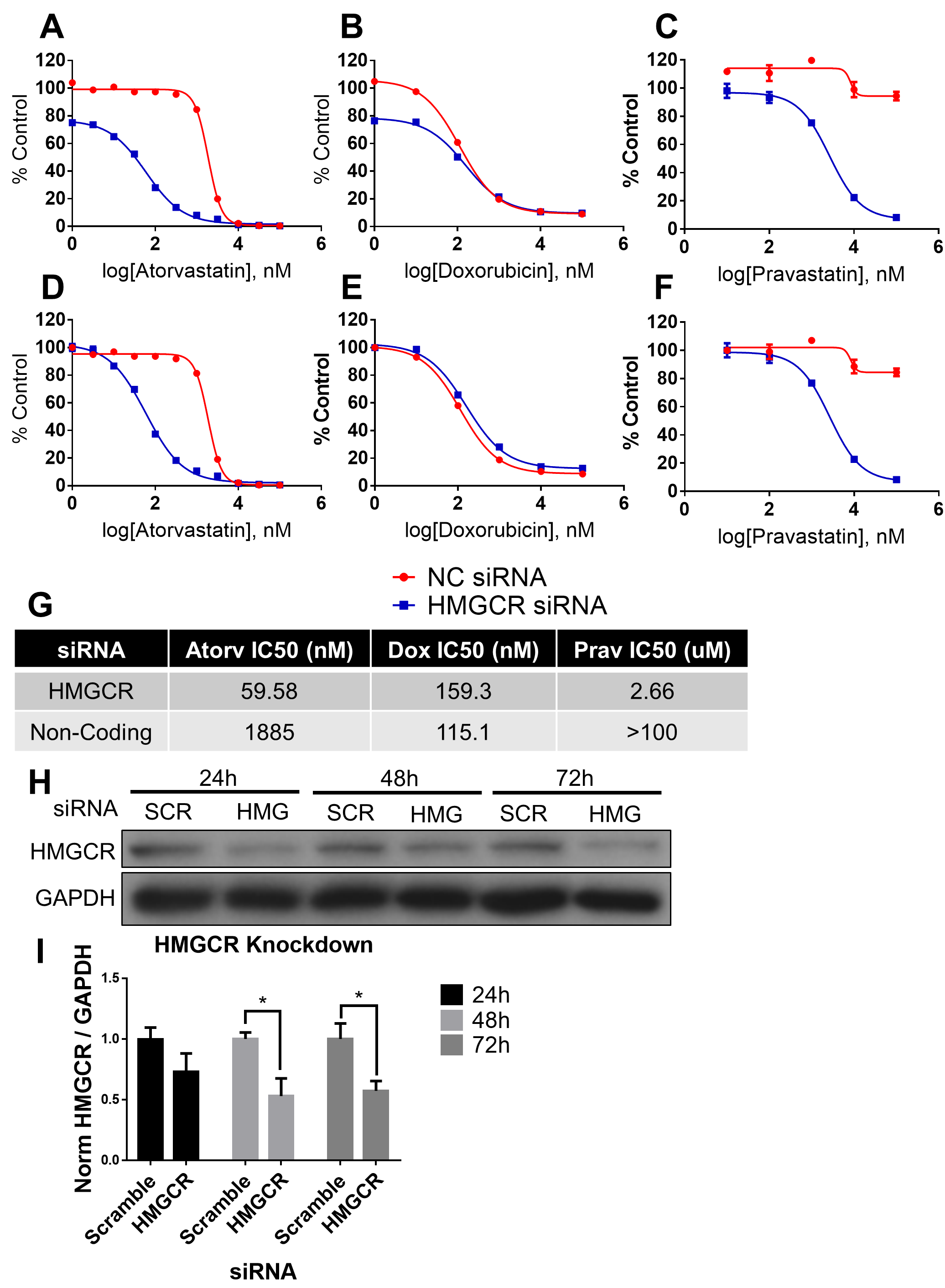

Supplement: S2 Fig — HMGCR was knocked down by siRNA treatment in MDA-MB-231 cells and cells were subsequently treated with (A,D) atorvastatin, (B,E) doxorubicin, or (C,F) pravastatin for 72 hours. (A-C) Data were normalized to the non-coding RNA control and then (D-F) further normalized to the lowest dose of drug used. (G) IC50 values for atorvastatin (Atorv), doxorubicin (Dox), and pravastatin (Prav) were calculated based on sigmoid curve fits to the dose response data. (H) HMGCR immunoblotting 24, 48, and 72 hours after siRNA knockdown with (I) quantification by densitometry. * P < 0.05. All data are representative of at least three independent experiments. (TIF) [file pone.0197422.s003.TIF]

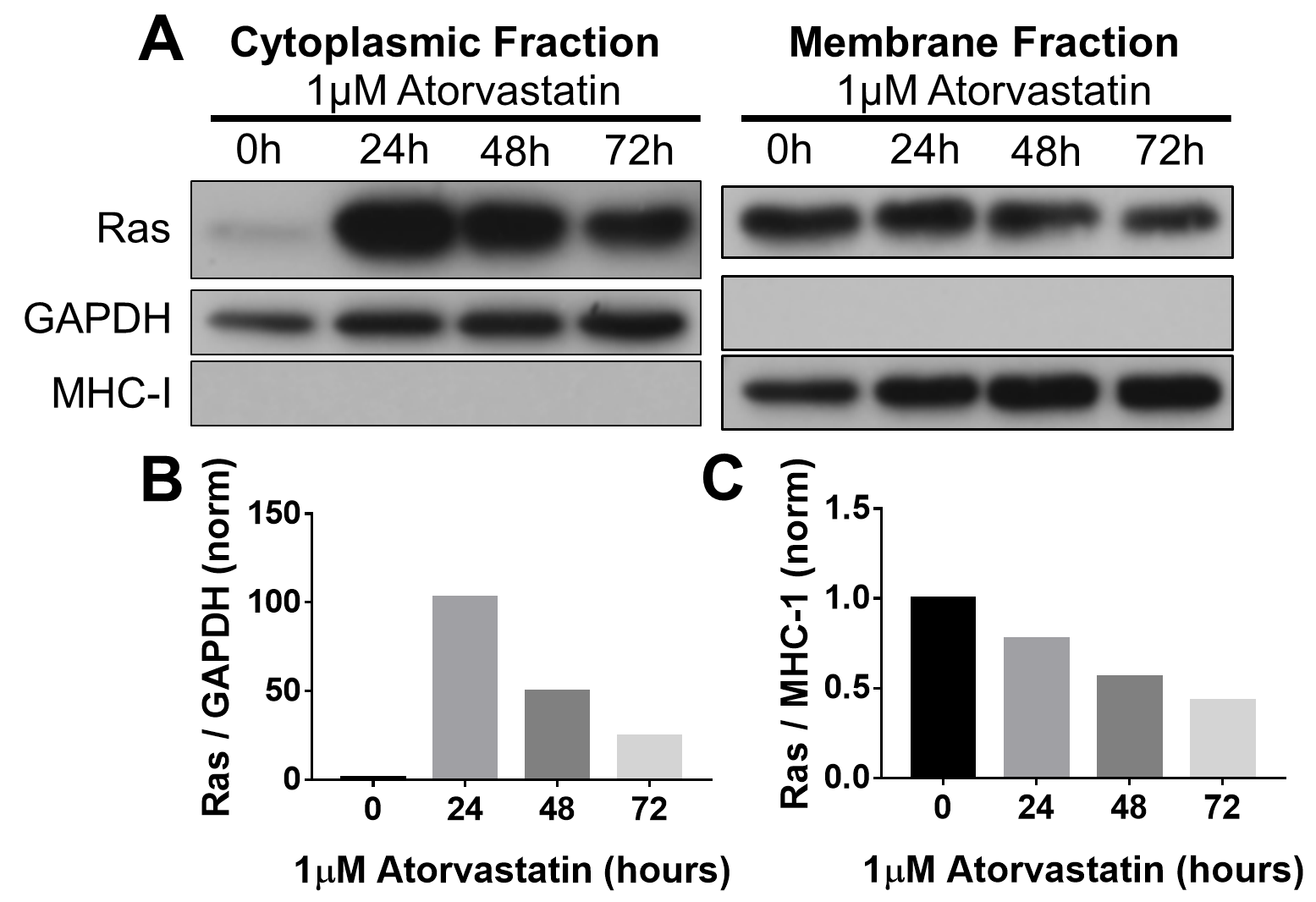

Supplement: S3 Fig — (A) MDA-MB-231 RFP cells were treated with 1μM atorvastatin for 0, 24, 48, or 72 hours and protein was collected in cytoplasmic and membrane fractions and probed by western blot. (B) Cytoplasmic Ras and (C) membrane Ras were quantified by densitometry. All data are representative of at least three independent experiments. (TIF) [file pone.0197422.s004.tif]

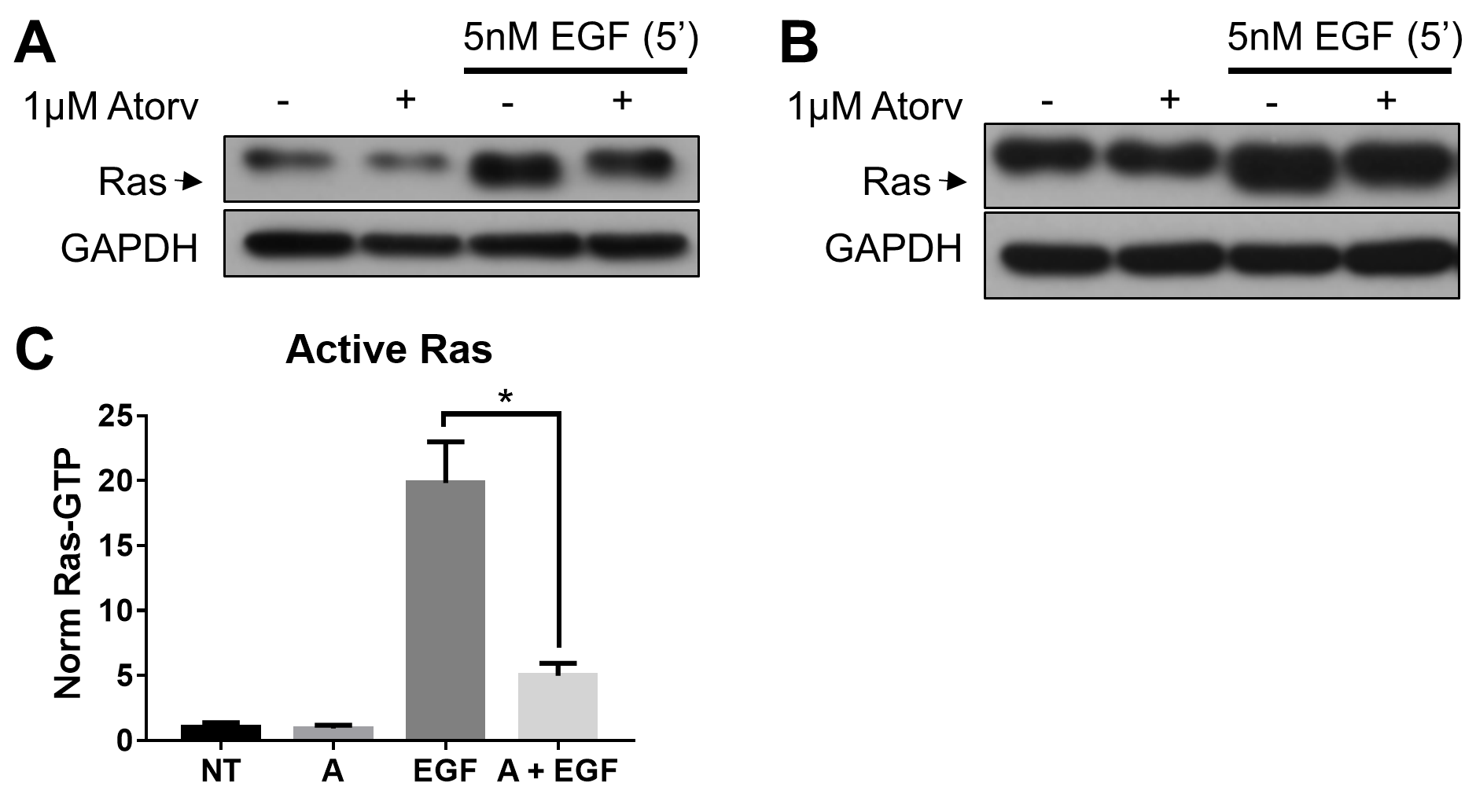

Supplement: S4 Fig — MDA-MB-231 RFP cells were treated with or without 1μM atorvastatin for 48 hours and then cells were stimulated with 5nM EGF for 5 minutes. Activated Ras (Ras-GTP) was isolated from cell lysates, (A,B) probed by western blot, and (C) quantified by densitometry of the faster mobility fraction. Atorv = Atorvastatin, NT = No treatment, A = 1uM Atorvastatin for 48 hours, EGF = 5nM EGF for 5 minutes. Error bars represent the SEM. * P < 0.05. All data are representative of at least three independent experiments. (TIF) [file pone.0197422.s005.TIF]

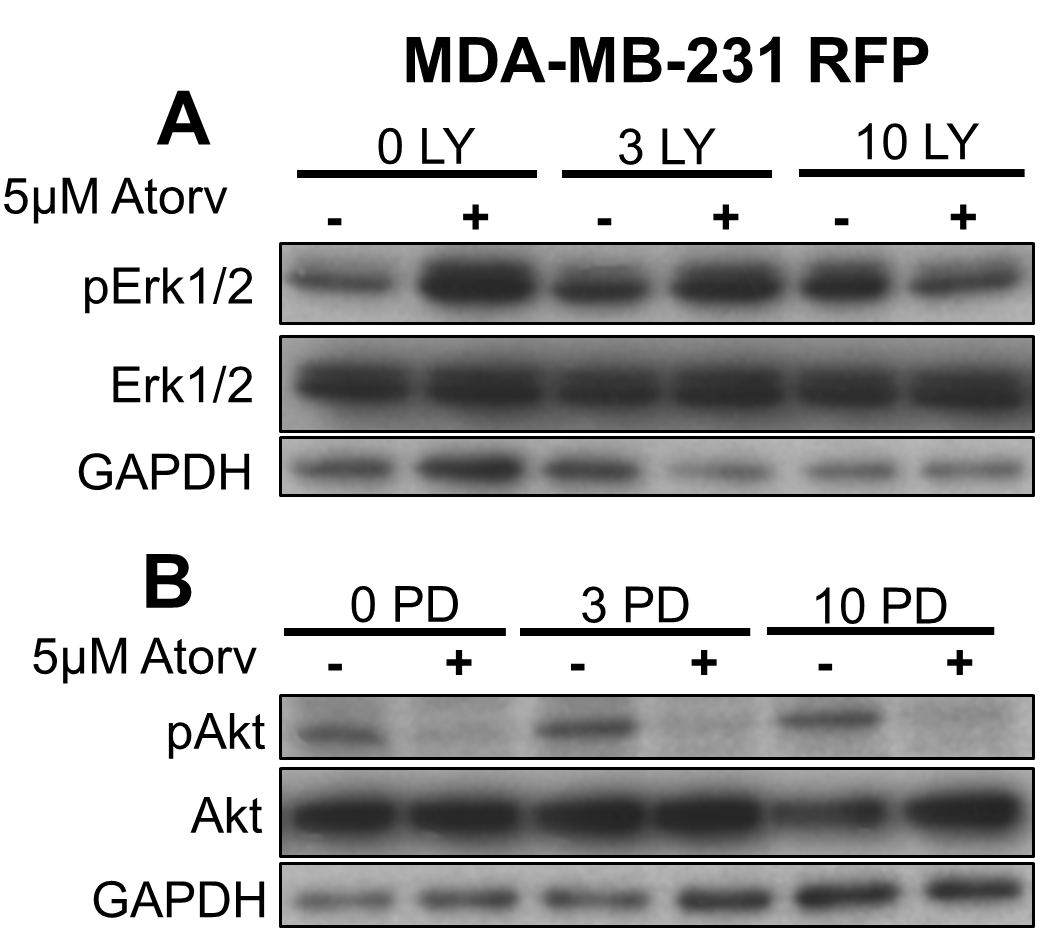

Supplement: S5 Fig — MDA-MB-231 RFP cells were treated with or without 5μM atorvastatin supplemented with (A) 0μM, 3μM, or 10μM LY294002 an inhibitor of PI3 kinase or (B) 0μM, 3μM, or 10μM PD98059 and inhibitor of MEK for 24 hours and (A) pErk and total Erk or (B) pAkt and total Akt were probed by western blot. Importantly, the distinction being made is with increasing doses of either LY294002 or PD98059 (comparing lanes 1, 3, and 5). The effect of atorvastatin treatment (comparing lanes 1 & 2, 3 & 4, and 5 & 6) on Akt and Erk phosphorylation is the same as shown in Fig 6. All data are representative of at least three independent experiments. (TIF) [file pone.0197422.s006.TIF]
